# Quantification of olanzapine and its three metabolites by liquid chromatography–tandem mass spectrometry in human body fluids obtained from four deceased, and confirmation of the reduction from olanzapine *N*-oxide to olanzapine in whole blood in vitro

**DOI:** 10.1007/s11419-023-00662-0

**Published:** 2023-03-30

**Authors:** Hideki Nozawa, Kayoko Minakata, Koutaro Hasegawa, Itaru Yamagishi, Naotomo Miyoshi, Masako Suzuki, Takuya Kitamoto, Minako Kondo, Kanako Watanabe, Osamu Suzuki

**Affiliations:** 1 Department of Legal Medicine, Hamamatsu University School of Medicine, 1-20-1 Handayama, Higashi-Ku, Hamamatsu, 431-3192 Japan; 2 Advanced Research Facilities and Services, Hamamatsu University School of Medicine, 1-20-1 Handayama, Higashi-Ku, Hamamatsu, 431-3192 Japan

**Keywords:** Olanzapine and its metabolites, In vitro conversion of olanzapine *N*-oxide to olanzapine, *N*-Desmethylolanzapine, 2-Hydroxymethylolanzapine, Authentic postmortem human specimens, Liquid chromatography, Tandem mass spectrometry

## Abstract

**Purpose:**

Quantification of olanzapine (OLZ) and its metabolites such as* N*-desmethylolanzapine (DM-O), 2-hydroxymethylolanzapine (2H-O) and olanzapine *N*-oxide (NO-O) in five kinds of human body fluids including whole blood by liquid chromatography (LC)–tandem mass spectrometry (MS/MS) has been presented; the quantification methods were carefully devised and validated using the matrix-matched calibration and standard addition methods.

**Methods:**

OLZ and its three metabolites were extracted from 40 μL each of body fluids by two-step liquid–liquid separations. The samples and reagents were pre-cooled in a container filled with ice for the extraction because of the thermal instability of OLZ and its three metabolites especially in whole blood.

**Results:**

The limits of quantification (LOQs) of OLZ and 2H-O were 0.05 ng/mL and those of DM-O and NO-O were 0.15 ng/mL in whole blood and urine, respectively. The concentrations of OLZ and its metabolites in heart whole blood, pericardial fluid, stomach contents, bile and urine were determined for two cadavers and those in whole blood and urine for the other two cadavers. The reduction from NO-O to OLZ was observed at 25 ℃ in whole blood in vitro.

**Conclusions:**

To our knowledge, this is the first report on the quantification of metabolites of olanzapine in the authentic human body fluids by LC–MS/MS as well as on the confirmation of in vitro reduction from NO-O to OLZ in whole blood that seems to have induced the quick decrease of NO-O.

**Supplementary Information:**

The online version contains supplementary material available at 10.1007/s11419-023-00662-0.

## Introduction

**Fig. 1:**
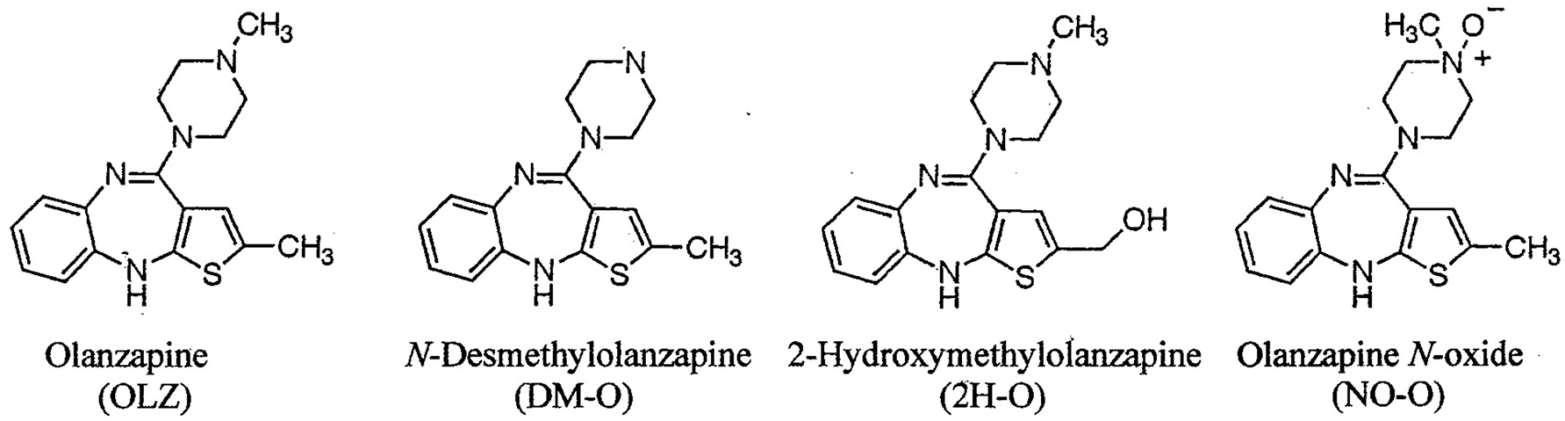
Structures of olanzapine (OLZ), *N*-desmethyl OLZ (DM-O), 2-hydroxymethyl OLZ (2H-O) and OLZ *N*-oxide (NO-O)

Olanzapine {2-methyl-4-(4-methyl-1-piperazinyl)-10*H*-thieno-[2,3-b] [1,5]benzodiazepine, OLZ} shown in Fig. 1, is an atypical antipsychotic agent, and has been being used in clinical practice since 1996. The in vitro metabolites of OLZ were produced first using human liver microsome in 1996 [1], where *N*-desmethyl OLZ (DM-O), 2-hydroxymethyl OLZ (2H-O) and OLZ *N*-oxide (NO-O) shown in Fig. 1 were analyzed based on radio activities of the respective ^14^C-compound. Subsequently, the in vivo disposition and biotransformation of OLZ were studied in six male healthy volunteers after a single dose of ^14^C-OLZ, where the tracing of radioactivity and liquid chromatography (LC)–tandem mass spectrometry (MS/MS) method were employed, and were confirmed not only the above three metabolites but also other metabolites such as olanzapine 10-*N*-glucuronide, olanzapine 4’-*N*-glucuronide and 2-carboxy OLZ [2]. In that work, however, the quantification of some of these metabolites was performed only by counting the intensity of ^14^C-radioactivities. These metabolites in human liver slices were also confirmed using LC–MS/MS method [3].

The quantification of OLZ metabolites in authentic human serum specimens by LC–MS/MS were published for DM-O [4–6], for DM-O with 2H-O [7] and for NO-O [8]. In most forensic investigations, however, the first-choice specimens are usually postmortem whole blood, in which the separation of serum or plasma is quite difficult, because of the hemolysis occurring in most postmortem whole blood specimens. Although quantification methods of OLZ in whole blood were briefly published before [9, 10], the quantification of DM-O, 2H-O and NO-O in whole blood has not been reported.

The stabilities of the metabolites in serum were described only on DM-O at ambient temperature after 2 or 3 h [4, 5], but those in whole blood have not been tried previously, although stability of only OLZ was examined [10].

In the present study, based on the stability tests, the quantification methods of OLZ and its three metabolites in five kinds of human body fluids were devised and validated, in which the matrix-matched calibration method was used for whole blood and urine, and the standard addition method [11–13] was used for pericardial fluid, stomach contents and bile because of unavailability of suitable blank matrices at our hands. The methods were applied to the quantification of OLZ and its three metabolites in the authentic specimens obtained from three cadavers who had been treated with OLZ therapeutically and from a deceased who had ingested a large amount of OLZ intentionally; only phase I metabolites of DM-O, 2H-O and NO-O were quantified, because the hydrolysis of the glucuronide compounds by glucuronidase at 37 ℃ affected the stabilities of these metabolites (data not shown).

## Case history

*Case 1*: A nearly 40-year-old man with a long history of paranoid schizophrenia was admitted to a hospital 12 days before his death. He ate his supper at 16:30 but he was found dead at 18:40. The autopsy was started 1.5 days after his death at our department. Around his nostril and mouth, there were vomit debris attached. The petechiae were observed at the conjunctiva palpebrarum. The internal examinations showed the congestion of several organs. The pharynx was plugged with a hard lump of toilet paper. The trachea was filled with stomach contents, which reached the tracheal bifurcation. His medications were noted to be OLZ, risperidone, bromazepam, biperiden and triazolam, and these medicines had been ingested at scheduled times, respectively. Five milligrams of OLZ had been ingested about 22 h before his death. The alcohol concentrations in heart whole blood and urine were undetectable and the concentrations of all the medicines were at the therapeutic levels. Cause of death was judged to be asphyxia after aspiration of stomach contents into the trachea. OLZ and its metabolites were quantified using his heart whole blood, pericardial fluid, stomach contents, bile and urine.

*Case 2*: A nearly 90-year-old woman was cared in a nursing home for the aged. Although her meal was limited to mixer meal, she was given a piece of kudzu mochi, a pudding-like arrowroot cake, by a stuff at around 14:45. She showed agonizing 10 min later, and was sent to a hospital by a rescue squad. A piece (6.5 × 4.1 × 1.7 cm) of kudzu mochi was sucked. Although her heart beat recovered once, she died at 16:51. The autopsy started about 3 days after her death. The petechiae were observed at the conjunctiva palpebrarum. The internal examinations showed the congestion of several organs. Cause of death was asphyxia due to the blockade of respiratory tract. She had been given 2.5 mg of OLZ to prevent her panic disorder and 20 mg of furosemide as a diuretic and hypotensive drug after breakfast. OLZ and its metabolites were quantified using her heart whole blood, pericardial fluid, stomach contents, bile and urine.

*Case 3*: A man in his thirties was found dead in a field 4 h after his neighbor saw a fire in the field. A melted polypropylene tank for kerosene, a lighter and others were scattered around his body. Autopsy was started at our department 3.5 days after discovery of his body. He was burned at the stages III and IV. Alcohol was not detected. Carbon monoxide hemoglobin saturation level was 28% in right heart blood. He had been diagnosed to be depression, and had been prescribed OLZ, bromazepam and clotiazepam until 1 month before his death. Afterwards, duloxetine, aripiprazole, clotiazepam were prescribed. The concentrations of these medicines were at the therapeutic levels. The cause of the death was burning himself using kerosene. OLZ and its metabolites were quantified for his heart whole blood and urine only.

*Case 4*: A woman in her thirties was found dead in her room with many empty blister packs. Her father saw her lastly 24 h before the discovery of her body. She had been diagnosed to be depression with suicide attempt, and had been prescribed OLZ (228 pills of the 5 mg-medicine were missing with no remains) and zolpidem (62 pills of 5 mg-medicine were missing with no remains). The lowest fatal concentrations of OLZ and zolpidem were 0.25 and 1.5 μg/mL in blood, respectively [14], and the determined concentrations in her blood were 6.81 ± 0.52 and 1.88 ± 0.11 μg/mL, respectively. The cause of the death was an acute intoxication with OLZ and zolpidem, and the autopsy was not performed because she was healthy except for her mental disease. OLZ and its metabolites were quantified for her heart whole blood and urine only that were collected about 20 h after the discovery of her body.

## Materials and methods

### Materials

OLZ and OLZ-*d*_8_ were obtained from Cayman Chemical (Ann Arbor, MI, USA); DM-O, 2H-O and NO-O, from Toronto Research Chemicals (Toronto, Canada); methanol and acetonitrile suitable for LC–MS, 1-chlorobutane suitable for amino acid analysis and other chemicals of analytical grade, from FUJIFILM Wako Pure Chemical Corporation (Osaka, Japan); blood and urine samples obtained from four healthy subjects (coauthors of this article) were used as blank samples (The Ethical Bureau of Hamamatsu University School of Medicine approved of using small amounts of blood and urine collected from the author and coauthors as blank samples in forensic analysis), and those spiked with several amounts of drugs were used as quality control samples.

The authentic specimens obtained from the cadavers were stored at – 80 ℃ until analyses.

### Standard solutions

Stock solutions of OLZ, OLZ-*d*_8_, DM-O, 2H-O and NO-O were prepared at 1 mg/mL in acetonitrile, and all stored at – 30 °C. OLZ-*d*_8_ was selected as the internal standard (IS). The stock solutions were diluted with acetonitrile as required. The IS was spiked to the samples at 10 ng/mL, respectively.

### Validation of the quantification methods

The quantification range was 0.05–10 for OLZ or 2H-O, and 0.15–30 ng/mL for DM-O or NO-O because ionization efficiency of the latter metabolites was lower than that of the former.

The matrix-matched calibration method was used for blood and urine. Calibrators were prepared at 0, 0.05, 0.1, 0.3, 1, 3 and 10 for OLZ or 2H-O (the former) and at 0, 0.15, 0.3, 0.9, 3, 9 and 30 ng/mL for DM-O or NO-O (the latter) in both blood and urine (*n* = 6 at each concentration). Quality control samples were prepared independently from the calibrators at 0.05, 1 and 10 for the former and 0.15, 0.3 and 30 ng/mL for the latter in both blood and urine (*n* = 3 at each concentration), and used for the calculation of accuracy, precision, recovery, matrix effect and limit of quantification (LOQ). Limit of detection (LOD) was calculated to be the concentration that gave signal to noise (S/N) ratio = 3 using three blank samples.

The standard addition method was used for pericardial fluid, stomach contents and bile of case 1, because suitable blank matrices were not available for the three specimens. At first, the concentration of analyte was roughly estimated according to reference [13]. For example, two samples, A and B were prepared as A; 100 μL of specimen + 1 μL of standard at 100 ng/mL and B; 100 μL of specimen + 1 μL of solvent. When the signals of A and B were 270 and 150, the concentration of the specimen was calculated to be 150/(270–150) = 1.25 ng/mL. Then each specimen was diluted with water to 1/10 or 1/100 (when required) so that concentration of OLZ or 2H-O was lower than 10 ng/mL, and that of DM-O or NO-O lower than 30 ng/mL, respectively. The calibrators were prepared by adding OLZ or 2H-O to the diluted specimen at 0, 0.1, 0.3, 1, 3 or 10 ng/mL, and DM-O or NO-O at 0, 0.3, 0.9, 3, 9 and 30 ng/mL (*n* = 6 at each concentration). For the intraday and interday repeatabilities, the calibration curves were measured five times; the concentration of analyte was determined as “D”, where the notifications (A’, B’, C and D) were described in reference [13]. Matrix effect values and recovery rates were calculated by adding the quality control (*n* = 3, respectively) to either the sample before the extraction (for example, A’: signal = 700), the sample after the extraction (B’: signal = 800) or the reconstitution solvent (C: signal = 500), where the amount of quality control (C) was selected to be the same amount of D (but its signal = 350). The percent matrix effect values and recovery rates were calculated as [(B’–D)/C] × 100 = [(800–350)/500] × 100 = 90 and [(A’–D)/(B’–D)] × 100 = [(700–350)/(800–350)] × 100 = 77.8, respectively.

### Extraction of OLZ and its metabolites from human body fluids

The extraction method was newly devised in this work and written as follows. Samples and reagents were placed in a cooler filled with ice for 15 min. To the 40-μL aliquot of each sample, 20 μL of water and 30 μL acetonitrile solution containing IS (at 10 ng/mL in the sample) were mixed. Then 170 μL of acetonitrile were added and vortexed for 1 min, and centrifuged at 10,000 × *g* for 4 min to precipitate impurities. The upper solution was transferred into a new tube, and 6 μL of saturated (NH_4_)_2_CO_3_ solution and 75 μL of 1-chlorobutane were added, vortexed for 1 min, and centrifuged at 10,000 × *g* for 2 min to separate organic layer from aqueous layer. Upper organic layer was transferred into a new tube and evaporated to about 80 μL using a centrifugal dryer (miVac Duo LV; Genevac Ltd, Ipswich, England), and 5 μL of it was used for LC–MS/MS analysis. (NH_4_)_2_CO_3_ solution was prepared daily. Vortex mixing, centrifugation and evaporation were performed at ambient temperature.

When the concentration was lower than LOQ, 400-μL sample was divided into 10 tubes of 40-μL sample, and each of them was processed to 80 μL as described above. To the total 800 μL of processed acetonitrile solution, 10 μL of saturated (NH_4_)_2_CO_3_ solution and 300 μL of 1-chlorobutane were again added, vortexed for 1 min, and centrifuged at 10,000 × *g* for 2 min to separate organic layer from aqueous layer. Upper organic layer was transferred into a new tube and evaporated to about 80 μL using a centrifugal dryer, and 5 μL of it was used for LC–MS/MS analysis.

### Instrumental conditions

LC–MS/MS was performed on an Exion LC instrument (SCIEX, Framingham, MA, USA) connected with an electrospray ionization QTRAP 5500 + MS/MS system (SCIEX) in the positive ion mode. A filter named SUMIPAX Filter PG-ODS (Sumika Chemical Analysis Service, Osaka, Japan) was attached to the precolumn before LC separation. The LC column for the chromatographic separation was TSK-GEL ODS-100 V (150 × 2.0 mm i.d., particle size 5 μm; Tosoh, Tokyo, Japan). The mobile phase consisting of 10% B (i.e., 90% A) was set at a flow rate of 200 μL/min, and then the gradient elution was started from 10 to 100% B over 10 min, switched to 100% B, held for 2 min, and returned to initial conditions over 8 min, where solvent A was pure water containing 10 mM ammonium acetate, and solvent B, 100% methanol. The MS/MS conditions were ion source temperature, 700 ℃; spray needle voltage, + 5.5 kV; sheath gas pressures, 30 units for gas 1 and 50 units for gas 2; curtain gas flow, 50 units. In the selected reaction monitoring (SRM) mode, the parent ions, product ions with their collision energies of OLZ and its metabolites are listed in Table S1, where each quantifier product ion was listed firstly among the four product ions. The collision energy and the transition were 31 V and *m/z* 321.2 → 261.1 for IS, respectively.

High-resolution LC–MS/MS was performed on an UltiMate 3000 coupled to a Thermo Scientific Q Exactive (quadrupole-Orbitrap) mass spectrometer (Thermo Scientific, Waltham, MA, USA) operated in positive ionization mode. Chromatographic separation was achieved with the same column under the same solvent conditions described above for LC–MS/MS performed on the QTRAP 5500 + MS/MS system. The MS or MS/MS conditions were spray voltage, 3.5 kV; capillary temperature, 250 ℃; heater temperature, 350 ℃; sheath gas flow rate, 50 units, and auxiliary gas flow rate, 15 units. Nitrogen was used for the collision-induced dissociation experiments. The instrument was calibrated every 24 h. The full MS resolution was 70,000 with scan range of *m/z* 220–2000, and MS/MS resolution was 17,500 with scan range of *m/z* 50–2000*.*

### Stability tests

Sodium ascorbate (Asc) was used to protect each sample from oxidation of OLZ in the previous work reported [9, 10]. In our preliminary work, however, 3 mM Asc in blood decreased the recovery of NO-O to less than 75%. Thus, Asc was not used for the detection of NO-O as well as for authentic specimens that may contain NO-O. To examine the in vitro effects of Asc and temperature on the stabilities of the four analytes, a 40-μL aliquot of blank blood or urine in a tube tightly capped was spiked with 4 μL of analyte in acetonitrile solution to make their concentrations to be 200 × LOQ for every analyte and then 4 μL of either 0 or 30 mM Asc solution (final concentration 3 mM) was added. They were incubated (*n* = 2, each) at 25 or 4 ℃ for 1, 2, 4 or 7 days and at − 30 ℃ for 3 months. In the case of NO-O in blood, they were also incubated at 25 ℃ for shorter periods such as 15, 45, 90, 150 or 240 min.

To examine freeze–thaw stability, samples were frozen at − 80 ℃ and thawed in water at 4 ℃ for 3 cycles.

After these treatments, IS was added at 10 ng/mL in blood or urine, and the concentrations of the target analyte as well as the other three analytes (OLZ, 2H-O and NO-O when DM-O was spiked) were quantified to examine the occurrence of interconversions among DM-O, OLZ, 2H-O and NO-O. Each detected molar amount of the analyte was compared with that in the freshly prepared analyte and was expressed as percentage value. In the stability test of NO-O in blood at 25 ℃, the test started with the sample plus reagents after preserving them in a cooler filled with ice (for 15 min), and incubated them at 25 ℃ for 15 to 240 min and for 1 to 7 days. Each amount of the analyte in the freshly prepared sample was considered to be 100%.

The extracted samples were preserved at 10 ℃ for 4 days or 4 ℃ for 7 days to examine the stability in an autosampler.

The stabilities of the authentic blood, urine and pericardial fluid of case 1 were also examined at 4 or 25 ℃ for 1 to 7 days and − 30 ℃ for 3 months.

## Results

### Selected reaction monitoring chromatograms and product ion spectra of OLZ and its metabolites

**Fig. 2:**
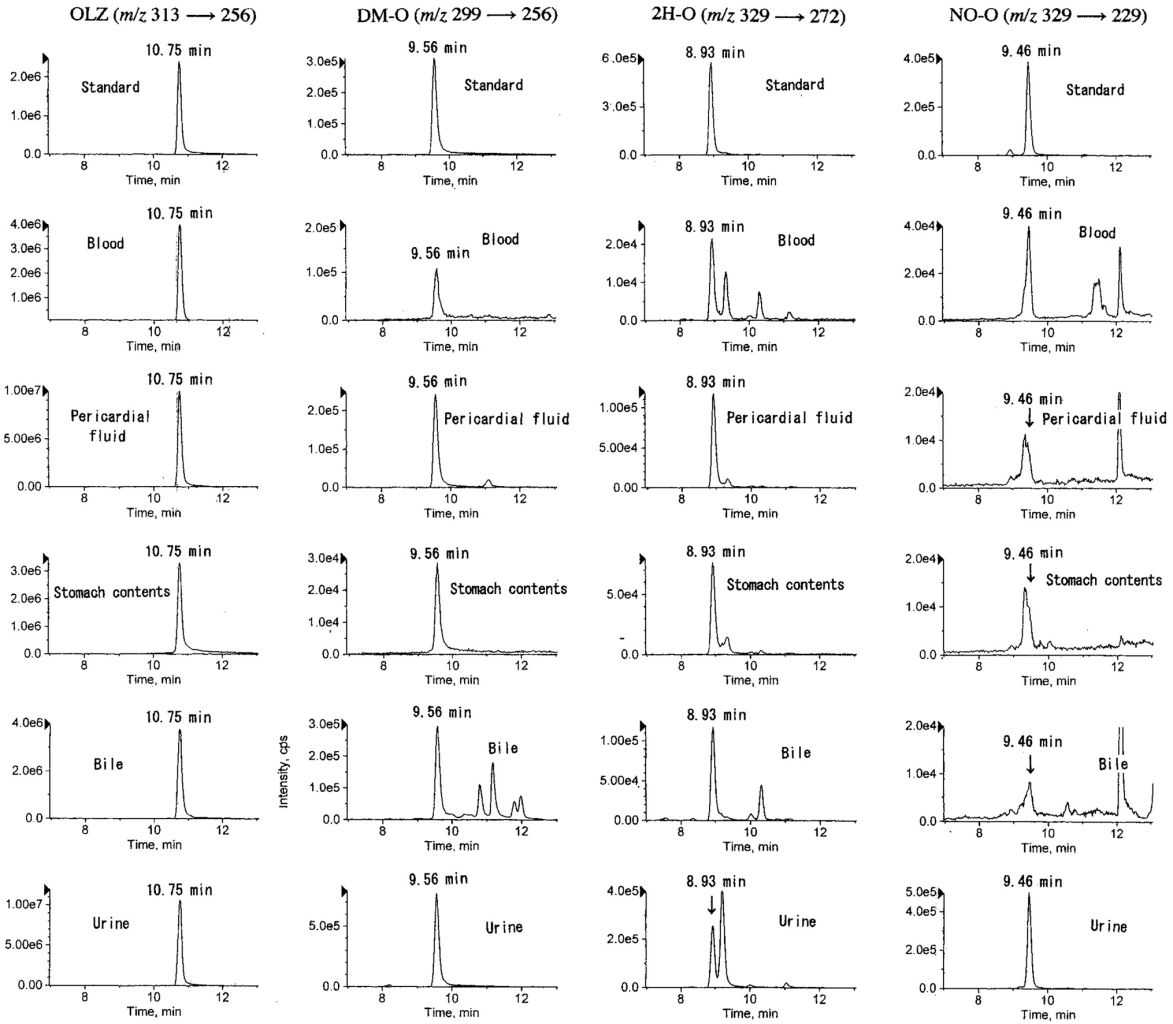
Selected reaction monitoring chromatograms by liquid chromatography (LC)–tandem mass spectrometry (MS/MS) for the detection of OLZ and its three metabolites, where reference standard, the extract from blood, pericardial fluid, stomach content, bile and urine specimens of case 1, respectively, were shown from the top to the bottom

The SRM chromatograms by LC–MS/MS for the detection of OLZ and its metabolites are shown in Fig. 2, where the reference standard extracted from water at 10 ng/mL for OLZ or 2H-O or at 30 ng/mL for DM-O or NO-O; and the chromatograms of extracts from whole blood, pericardial fluid (diluted to 1/2), stomach contents, bile (diluted to 1/10) and urine (diluted to 1/4) specimens in case 1 are also shown from the top to the bottom. In this figure, the retention times were almost the same in the same analyte with different matrices within the error of 0.01 min. The extracts from blank whole blood and urine did not show any peaks that interfered with the detection of the analytes. Since there are several isomers for hydroxy compounds and *N*-oxides of OLZ, these compounds cannot be identified without confirming the isomers.

**Fig. 3:**
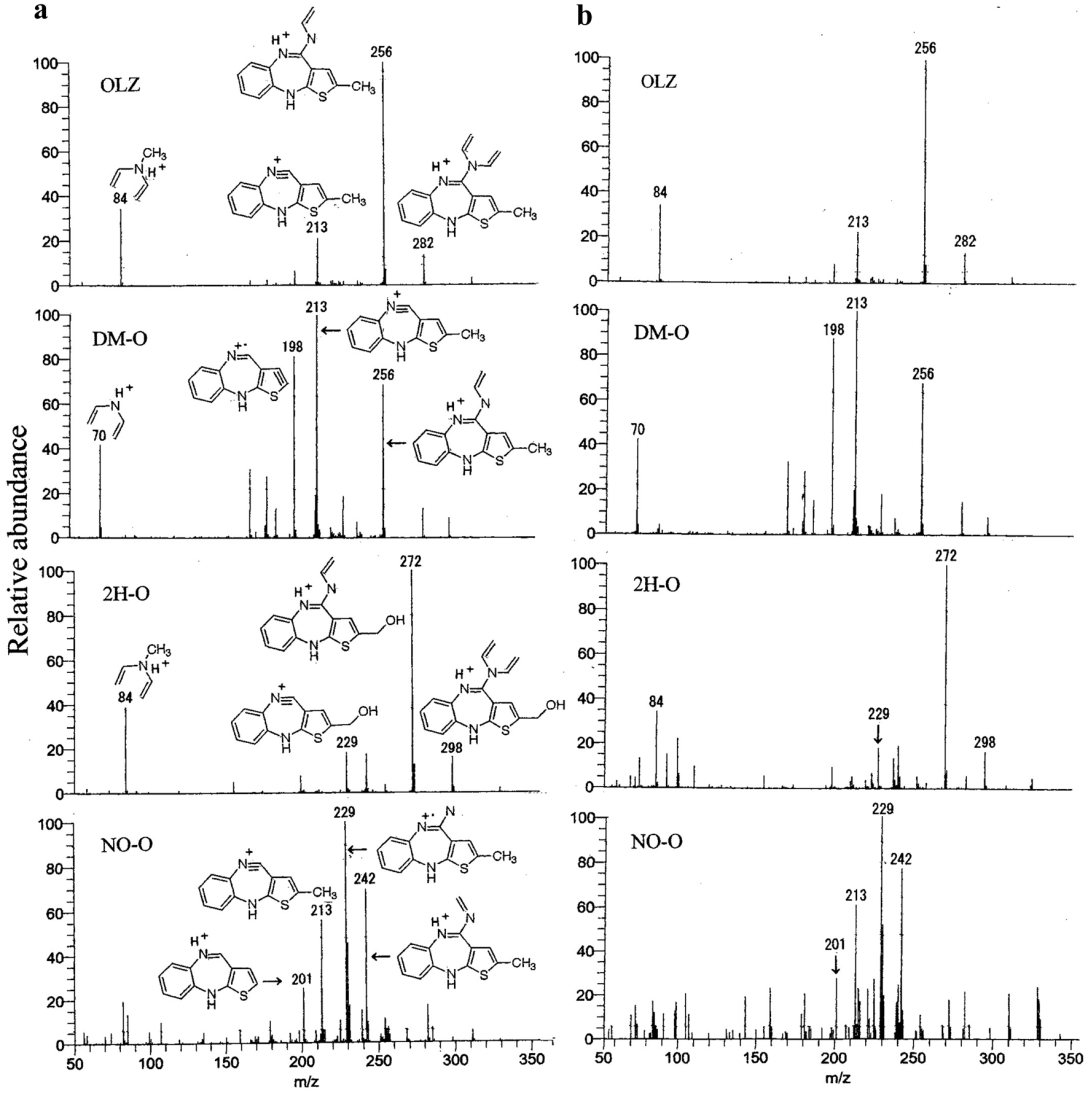
Product ion spectra detected by LC–Orbitrap–MS/MS and structures of product ions for OLZ and its three metabolites where the reference standards were shown in (**a**) the left panels and the extract from urine of case 1 (concentrated to twofold) in (**b**) the right panels

Figure 3 shows the product ion spectra for OLZ and its metabolites detected by LC–Orbitrap-MS/MS. The reference standard extracted from water at 200 ng/mL is shown in the left panel (Fig. 3a) and the extract from the urine specimen in case 1 in the right panel (Fig. 3b). More probable figures were described in Fig. 3a because mass spectra cannot suggest structures such as positions of functionals or the difference between two double bonds and one triple bond with one single bond or others. The *m/z* values and their relative ratios of product ions in the urine of case 1 agreed quite well with those in the reference standards. Furthermore, the maximum error between the *m/z* values of the product ions in the urine and those of the theoretical values calculated from the estimated structures was only 11 ppm as listed in Table S1.

The relative peak height ratios of four principal product ions in the reference standard solution and those in the urine of case 1 were determined by LC–MS/MS, and are listed in Table S2 by taking the highest product ions to be 100. The relative ratios of the product ions of reference standards and those of urine specimen agreed well with each other.

The observed retention times of the peaks in Fig. 2, the product ion spectra of the urine in Fig. 3 and the relative peak height ratios in the urine in Table S2 agreed well with those of the reference standards respectively, confirming that the peaks from the authentic urine specimen were due to OLZ and its metabolites.

### Reliability of the matrix-matched calibration method for OLZ and its metabolites in whole blood and urine

The concentration linearities of OLZ and its metabolites using the conventional matrix-matched calibration method were examined by spiking the compounds to blank whole blood and urine at 0, 0.05, 0.1, 0.3, 1.0, 3.0 and 10 ng/mL for OLZ or 2H-O and at 0, 0.15, 0.3, 0.9, 3.0, 9.0 and 30 ng/mL for DM-O or NO-O in whole blood and urine (*n* = 6 at each concentration), respectively. The regression equations for the calibration curves are listed in Table S3, where the correlation coefficients were 0.994–0.998. The LODs are also listed there. The precisions and the accuracies were assessed by analyzing quality controls spiked with OLZ or 2H-O at 0.05, 1.0 and 10 ng/mL and spiked with DM-O or NO-O at 0.15, 3.0 and 30 ng/mL, which are based on measurements three times a day and on measurements on three different days. The accuracy values were 83.9–114% in blood and 75.6–118% in urine and the precision values were not greater than 14.1% in both matrices for intraday and interday measurements as listed in Table S4. The recovery values in the quantification ranges were 75.0–107% (*n* = 3 at each concentration) and the matrix effect values were 77.0–107% (*n* = 3 at each concentration). These validation data could be considered to be within the acceptable range for quantification [15].

### Reliability of the standard addition method for OLZ and its metabolites in pericardial fluid, stomach contents and bile

The concentration linearities of OLZ and its metabolites using the standard addition method were examined by spiking OLZ or 2H-O to suitably diluted or not diluted specimens at 0, 0.1, 0.3, 1.0, 3.0 and 10 ng/mL, and DM-O or NO-O at 0, 0.3, 0.9, 3.0, 9.0 and 30 ng/mL. The regression equations for the standard addition calibration curves are listed in Table S3 (lower panel), where the correlation coefficients were 0.992–0.999. The LODs in the solutions are also listed in the table.

Intraday and interday determinations of OLZ and three metabolites in three kinds of body fluids were repeated five times as listed in Table S5. The repeatability values, expressed as relative standard deviations (RSDs), were not greater than 23.6%. The recovery rates were 78.0–96.7% with the maximum RSD of 20.0% and the matrix effect values were 76.1– 92.5% with the maximum RSD of 14.4% as listed in Table S5. These validation data could be considered to be within the acceptable range for quantification [15].

## Results of stability tests

The results of in vitro stability tests of the analytes spiked into urine with or without Asc at 25 ℃ are listed in the Table 1. Small amounts of 2H-O and NO-O were produced from OLZ in urine at 25 ℃ after several days. All analytes in urine tended to decrease until 7 days without Asc at 25 ℃. When 3 mM Asc was added to the urine spiked with either OLZ, DM-O or 2H-O, they were stable (85 > %) after the incubation at 25 ℃ up to 7 days, where appreciable production of 2H-O and NO-O from OLZ was not observed. At 4 ℃, all analytes, NO-O, OLZ, DM-O and 2H-O, in urine were stable without Asc up to 7 days (data not listed).

**Table 1:** In vitro 7-day and 240-min stabilities (%) for OLZ and its metabolites in urine or whole blood added or not added with sodium ascorbate (Asc)

Seven-day stabilities								
Original analyte	Sample	*Tem *(°C)	*Asc* (mM)	Detected analyte	Duration (day)			
					0	1	4	7
NO-O	Urine	25	0	NO-O	100	95.0	82.0	60.0
NO-O	Blood	25	0	NO-O	98.5	25.8	16.0	2.9
				OLZ	1.4	17.9	12.6	4.2
				DM-O	0.1	2.0	1.3	0.6
NO-O	Blood	4	0	NO-O	98.5	86.9	84.9	84.9
				OLZ	1.4	2.3	2.8	3.2
				DM-O	0.1	0.4	0.6	0.6
OLZ	Urine	25	0	OLZ	100	90	82	70
				2H-O	0	0.4	1.9	3.8
				NO-O	0	0	1.0	2.9
OLZ	Urine	25	3	OLZ	100	102	98	95
OLZ	Blood	25	0	OLZ	100	57	48	27
OLZ	Blood	25	3	OLZ	100	77	53	32
OLZ	Blood	4	0	OLZ	100	110	100	85
DM-O	Urine	25	0	DM-O	100	98	85	66
DM-O	Urine	25	3	DM-O	100	99	89	90
DM-O	Blood	25	0	DM-O	100	50	32	20
DM-O	Blood	25	3	DM-O	100	78	64	27
DM-O	Blood	4	0	DM-O	100	85	80	77
2H-O	Urine	25	0	2H-O	100	103	95	86
2H-O	Urine	25	3	2H-O	100	101	96	99
2H-O	Blood	25	0	2H-O	100	83	66	40
2H-O	Blood	25	3	2H-O	100	100	98	65
2H-O	Blood	4	0	2H-O	100	105	90	101

Stabilities for 240-min										
Original analyte	Sample	*Tem *(°C)	*Asc* (mM)	Detected analyte	Duration (min)					
					0	15	45	90	150	240
NO-O	Blood	25	0	NO-O	98.5	91.9	86.9	68.6	58.9	48.3
				OLZ	1.4	2.1	4.4	5.8	6.3	12.5
				DM-O	0.1	0.2	0.4	0.6	0.9	1.3

The concentrations of NO-O, OLZ, DM-O and 2H-O were 200 x LOQ (ng/mL)

*Tem *temperature, *Asc *sodium ascorbate

The results of stability tests for the analytes spiked into blank whole blood with or without Asc at 25 or 4 ℃ are listed in Tables 1 and 2. Concerning OLZ, DM-O and 2H-O, the addition of 3 mM Asc was found to give better results than without Asc for the protection of them, but the addition of Asc could not prevent the decrease efficiently at 25 ℃. Concerning the NO-O spiked, OLZ and DM-O were detected even at the beginning of the stability test, and hence the stability tests were started using samples and reagents placed in a cooler (time 0 min after cooling for 15 min). After 240 min incubation at 25 ℃, NO-O decreased to 48.3% and OLZ and DM-O increased to 12.5 and 1.3%, respectively, as listed in the lower panel of Table 3. NO-O continued to decrease afterwards, but OLZ and DM-O showed their maximum values, 17.9 and 2.0%, after 1 day and then decreased, respectively.

**Table 2:** In vitro stabilities (%) of OLZ and its three metabolites at 25 or 4 ℃ in the authentic urine, whole blood and pericardial fluid specimens of case 1

			Urine				Whole blood				Pericardial fluid			
Original analyte	*Tem *(°∁)	Detected analyte	Duration (day)				Duration (day)				Duration (day)			
			0	1	4	7	0	1	4	7	0	1	4	7
NO-O	25	NO-O	100	89	85	68	100	27	16	15	100	85	62	57
	4		100	103	88	91	100	95	92	86	100	92	90	88
OLZ	25	OLZ	100	96	88	77	100	76	60	52	100	98	87	73
	4		100	89	103	94	100	95	98	88	100	105	93	90
DM-O	25	DM-O	100	91	87	75	100	61	41	37	107	110	75	53
	4		100	98	96	87	100	93	92	86	100	92	91	87
2H-O	25	2H-O	100	104	98	87	100	83	70	60	100	104	100	110
	4		100	106	99	98	100	98	103	95	100	95	102	98

All analytes in whole blood incubated at 4 ℃ were much more stable than those at 25 ℃ and hence samples and reagents were placed in a cooler just before the extraction; vortexing and centrifugation were performed at room temperature. All analytes in blood and urine were stable up to 3 months at –30 ℃.

The analytes in blood were quite labile at 25 ℃, and hence freeze–thaw experiment was performed between − 80 ℃ and 4 ℃ (placed in water at 4 ℃). All the analytes in whole blood and urine were stable for 3 cycles of the freeze–thaw experiment.

The extracted analytes dissolved in acetonitrile and 1-chlorobutane were stable for up to 4 days at 10 ℃ or for 7 days at 4 ℃.

The results of stability tests at 25 or 4 ℃ in the authentic urine, whole blood and pericardial fluid of case 1 are listed in Table 2. The results of stability tests at 25, 4 or − 30 ℃ in the authentic urine and whole blood were similar to those of the samples spiked. In case of NO-O in the authentic whole blood specimen preserved at 25 ℃, the quick decrease of NO-O was observed but the increase of OLZ and DM-O could not be observed. The stabilities of analytes in the matrices were in the order of urine ≥ pericardial fluid > blood.

### Quantification of OLZ and its metabolites in all authentic specimens collected

The concentrations of OLZ and its metabolites in the specimens of cases 1 to 4 are listed in Table 3. The ratios (%) of the concentrations of metabolites to that of OLZ are also listed. The specimens were diluted suitably (or concentrated up to 10 times), because the quantification ranges were 0.05 (or 0.1 in the standard addition method) – 10 ng/mL for OLZ and 2H-O, and 0.15 (or 0.3)– 30 ng/mL for DM-O and NO-O. Some metabolites could not be quantified even after 10-time concentration and marked as “–^a^” in the table. The detected concentrations of analytes varied greatly from case to case. However, some similarity and dissimilarity were observed as to the ratios of metabolites to OLZ. First, the ratio of DM-O/concentrations much higher than that of 2H-O/OLZ or NO-O/OLZ in bile samples of cases 1 and 2. Second, the ratios of metabolites/concentrations large in cases 1 and 3 indicating that the metabolism of OLZ proceeded to certain extents, but was minute in case 4 suggesting that the metabolism stopped shortly after the ingestion.

## Discussion

Table 1 shows that 2H-O and NO-O were produced from OLZ in urine at 25 ℃. Atmospheric oxygen may be related to the oxidation of OLZ because 2H-O and NO-O were produced from OLZ in water nearly to the same extent (data not shown).

Very quick decrease of imipramine *N*-oxide due to in vitro interconversions was reported previously in the homogenates of rat blood, liver, kidney and others [16] and also the homogenates of livers of rat, horse and pig species [17], where its *N*-oxide decreased to 22%, and imipramine and desmethyl imipramine increased from 0 to 54% and from 0 to 3% after 120 min at 37 ℃ in rat liver, respectively [16]. The similar interconversions were also observed in the present stability test of human whole blood as listed in Table 1; NO-O decreased to 48.3%, and OLZ and DM-O increased to 12.5 and 1.3%, respectively, after 240 min at 25 ℃. The production of OLZ and DM-O from NO-O in the whole blood of the present work at 25 ℃ might be caused by oxyhemoglobin, reduced cytochrome C, other hemoproteins and/or minutely by Fe^2+^ chelated with citrate and others [16]. Therefore, whole blood and reagents were stored in a cooler.

Acetonitrile denatured proteins and hence the drugs combined to them were released into acetonitrile solution. After centrifugation, the denatured proteins were eliminated from the acetonitrile solution by the first-step extraction; and still solved components such as Fe^2+^, citrate and others were eliminated together with water by the second-step extraction. After these extractions, OLZ and its metabolites were dissolved in the mixture of acetonitrile and 1-chlorobutane. The extracted analytes were stable at least for 4 days at 10 ℃ or 7 days at 4 ℃ as described in the above results of stability tests.

Biological functions of OLZ were compared with those of its three metabolites using mice, and only NO-O among the three metabolites could block partially apomorphine-induced climbing behavior observed in OLZ [18]. There is a possibility that the reason for the observed blocking phenomenon by NO-O is due to OLZ because OLZ could be produced from NO-O at 25 ℃ in blood as listed in Table 1 (lower panel).

NO-O, DM-O and 2H-O were only decreased, but small amounts of OLZ were produced from NO-O when they were quantified separately as listed in Table 1. However, this increase of OLZ could not be observed in the blood of case 1 as listed in Table 2. Since in the real samples the ratio of NO-O/concentrations 0.17 shown in Table 3, the increment of OLZ from NO-O was much smaller than the decrement of OLZ. Therefore, all the analytes were decreased in the whole blood of case 1 without inconsistency. The instability of OLZ was shown in the works [9, 10] but the causes of instability of not only OLZ but also three metabolites had not been discussed yet. The three metabolites were produced from OLZ by *C*-oxidation and *N*-oxidation even in pure water. Since sulfur is easily oxidized, the oxidation of sulfur by some enzymes or unknown molecules in blood at 25 ℃ may be one of the reasons why OLZ and three metabolites decreased.

**Table 3:** Concentrations of OLZ and its three metabolites (ng/mL) and concentration ratios of metabolites to that of OLZ in human body fluids of the four cases

Concentration (ng/mL)							Ratio to OLZ		
Case		OLZ	DM-O	2H-O		NO-O	DM-O/OLZ	2H-O/OLZ	NO-O/OLZ
1	Right heart blood	25.9 ± 2.9	16.9 ± 1.4	0.45 ± 0.06		4.33 ± 0.35	0.65	0.017	0.17
1	Pericardial fluid	70.8 ± 7.1	39.6 ± 3.4	2.82 ± 0.33		0.97 ± 0.08	0.56	0.040	0.014
1	Stomach contents	16.9 ± 0.7	3.10 ± 0.30	1.40 ± 0.22		0.66 ± 0.05	0.18	0.083	0.039
1	Bile	200 ± 18	346 ± 40	20.8 ± 2.3		6.30 ± 1.07	1.73	0.10	0.032
1	Urine	166 ± 17	271 ± 18	12.9 ± 1.2		132 ± 11	1.63	0.078	0.80
2	Right heart blood	0.329 ± 0.047	0.026 ± 0.002	0.007 ± 0.002		–^a^	0.079	0.021	–
2	Pericardial fluid	0.117 ± 0.010	0.019 ± 0.003	–		0.024 ± 0.002	0.16	–	0.21
2	Stomach contents	0.130 ± 0.020	0.025 ± 0.003	–		0.032 ± 0.003	0.19	–	0.25
2	Bile	0.053 ± 0.007	0.043 ± 0.005	–		–	0.81	–	–
2	Urine	0.079 ± 0.014	–	–		–	–	–	–
3	Blood	0.125 ± 0.015	0.538 ± 0.086	–		–	4.30	–	–
3	Urine	0.71 ± 0.07	1.10 ± 0.07	0.025 ± 0.003		0.63 ± 0.05	1.55	0.035	0.89
4	Blood	6810 ± 520	1100 ± 120	73.8± 6.5		77.7 ± 8.6	0.16	0.011	0.011
4	Urine	3600 ± 250	437 ± 51	32.0 ± 2.3		15.3 ± 2.3	0.12	0.009	0.004

The “blood” means whole blood

–^a^Unquantifiable

As listed in Table 3, the concentrations of analytes in case 2 were much lower than those in case 1, although the dose of case 2 was about a half of that of case 1. There are differences due to the age of victims, the duration after ingestion of OLZ and the period between the death and the autopsy (postmortem interval) in the two cases: nearly 40 years old, 22 h after the ingestion and the postmortem period of 1.5 days in case 1, whereas nearly 90 years old, 7 h after the ingestion and the postmortem period of 3 days in case 2. However, even with the consideration of such condition differences between the two cases, the vast differences in OLZ and its metabolite levels found between the two cases are not explicable. There may be inexplicable individual difference in the two cases. Dusci et al. [19] also reported that antemortem concentrations of OLZ treated with the same daily doses varied more than ten times even in groups of therapeutically treated patients.

The analyte stability in urine was higher than that in blood (Table 2) but the concentrations of analytes in urine cannot be used as the indicator of toxicity, because the volumes of urine in cadavers varied from 0 to 400 mL, while the volume of blood was roughly 5 L in subjects of 65 kg and its concentration can be used as the indicator. The concentrations of analytes in pericardial fluid can be used as a rough indicator of toxicity because analytes stability was higher than that in blood and the volume of pericardial fluid is roughly 20–50 mL in normal subjects, although the number of the examined cases was only two.

## Conclusions

A sensitive method was established for the quantification of OLZ and its three metabolites in several human body fluids using LC–MS/MS. OLZ and its three metabolites were labile in whole blood but they were relatively stable in urine and pericardial fluid at ambient temperature. Therefore, preservation of blood and reagents in a cooler is recommended before the extraction procedure. The LOQs of OLZ and 2H-O were 0.05 ng/mL and those of DM-O and NO-O were 0.15 ng/mL in both blood and urine. The reduction of NO-O to OLZ was observed in whole blood in vitro for the first time, and the phenomenon seems to be the main cause of quick decrease of NO-O at 25 ℃. OLZ and its three metabolites in the authentic human specimens obtained from four deceased were quantified, and both the matrix-matched calibration and standard addition methods used were fully validated.

## Supplementary Information

Below is the link to the electronic supplementary material.

Supplementary file1 (DOCX 51 KB)
