# Supplementary material for: Quantification of olanzapine and its three metabolites by liquid chromatography–tandem mass spectrometry in human body fluids obtained from four deceased, and confirmation of the reduction from olanzapine N-oxide to olanzapine in whole blood in vitro
Source: Forensic Toxicol. 2023 Mar 30;41(2):318–28. doi: 10.1007/s11419-023-00662-0 (PMC10310574; doi:10.1007/s11419-023-00662-0)
Supplement: Supplementary file 1 — Supplementary file1 (DOCX 51 KB) [file 11419_2023_662_MOESM1_ESM.docx]

Electronic supplementary material

**Quantification of olanzapine and its three metabolites by liquid chromatography-tandem mass spectrometry in human body fluids obtained from four deceased, and confirmation of the reduction from olanzapine *N*-oxide to olanzapine in whole blood in vitro**

**Hideki Nozawa^1^**・**Kayoko Minakata^1^**・**Koutaro Hasegawa^1^**・**Itaru Yamagishi^1^**・**Naotomo Miyoshi^1^**・ **Masako Suzuki^1^**・**Takuya Kitamoto^2^**・**Minako Kondo^2^**・**Kanako Watanabe^1^**・**Osamu Suzuki^1^**

^1^ Department of Legal Medicine, Hamamatsu University School of Medicine, 1-20-1 Handayama, Higashi-ku, Hamamatsu 431-3192, Japan

^2^ Advanced Research Facilities and Services, Hamamatsu University School of Medicine, 1-20-1 Handayama, Higashi-ku, Hamamatsu 431-3192, Japan

Hideki Nozawa (corresponding author)

(tel) +81-53-435-2239, (fax) +81-53-435-2858,

(e-mail) hnozawa@hama-med.ac.jp

**Table S1** Accurate mass data showing elemental compositions, retention times, precursor ions and diagnostic product ions with their mass errors in parentheses (ppm) for olanzapine and its three metabolites in urine detected by liquid chromatography (LC)–Orbitrap tandem mass spectrometry (MS/MS)

Analyte Elemental Retention Precursor ion Collision Diagnostic product ions

composition time in *m/z* observed voltage in *m/z* observed

(min) (mass error, ppm) (V) (mass error, ppm)

OLZ C17H20N4S 10.75 313.1482 (0) 31 256.0902(0), 282.1057(-1)

213.0482(0), 84.0814(+7)

DM-O C16H18N4S 9.55 299.1323(-1) 35 256.0904(0), 213.0482(0)

198.0248(+1), 70.0659(+11)

2H-O C17H20N4OS 8.93 329.1425 (-2) 33 272.0852(0), 298.1007(-1)

229.0433(+1), 84.0814(+7)

NO-O C17H20N4OS 9.46 329.1425(-2) 25 229.0664(-2), 242.0748(+1)

213.0482(0), 201.0483(+1)

*OLZ* olanzapine, *DM-O N*-desmethyl olanzapine, *2H-O* 2-hydroxymethyl olanzapine, *NO-O* *N*-oxide olanzapine

**Table S2** Relative peak height intensities of the product ions of OLZ and its metabolites in the reference standard solution and urine of the victim in case 1 taking the intensity of the highest ion to be 100 detected by LC–MS/MS

Analytes Protonated Product ion (*m/z*)

molecular ion (*m/z*)

Specimen Percent product ion intensity

OLZ (313) (256) (282) (213) (84)

Standard 100 11.3±0.2 7.7±0.1 19.8±0.9

Urine 100 11.7±0.2 8.3±0.4 20.1±0.3

DM-O (299) (256) (213) (198) (70)

Standard 100 89.4±0.5 31.2±0.4 19.9±0.3

Urine 100 88.1±0.8 30.2±0.5 19.0±0.1

2H-O (329) (272) (298) (229) (84)

Standard 100 11.8±0.2 7.4±0.1 20.7±0.3

Urine 100 11.2±0.2 6.8±0.1 19.2±0.4

NO-O (329) (229) (242) (213) (201)

Standard 100 63.7±1.2 15.5±3.9 22.7±2.3

Urine 100 60.4±0.9 14.2±0.3 22.7±1.6

**Table S3** Regression equations, correlation coefficients, limits of quantification (LOQ), limits of detection (LOD) for OLZ and its metabolites in whole blood, urine and other three specimens measured by the conventional matrix-matched calibration method (upper panel) and the standard addition method (lower panel)

Matrix-matched calibration

Analyte LOQ – 200 x LOQ equation Correlation LOD

(ng/mL) coefficient (ng/mL)

**Whole blood spiked**

OLZ 0.05 – 10  *y =* 0.2122 *x* + 0.0026 0.994 0.02

DM-O 0.15 – 30  *y =* 0.0086 *x* + 0.0000 0.998 0.05

2H-O 0.05 – 10  *y =* 0.0606 *x* + 0.0009 0.998 0.02

NO-O 0.15 – 30  *y =* 0.0107 *x* - 0.0001 0.997 0.15

**Urine spiked**

OLZ 0.05 – 10  *y =* 0.1696 *x* + 0.0068 0.996 0.02

DM-O 0.15 – 30  *y =* 0.0087 *x* + 0.0009 0.998 0.05

2H-O 0.05 – 10  *y =* 0.0750 *x* + 0.0011 0.996 0.02

NO-O 0.15 – 30  *y =* 0.0109 *x* + 0.0007 0.997 0.05

Standard addition calibration

Analyte Spiked range equation Correlation LOD

(dilution rate) (ng/mL) coefficient (ng/mL)

**Pericardial fluid in case 1**

OLZ (1/10) 0.1 – 10  *y =* 0.1430 *x* + 0.9833 0.995 0.02

DM-O (1/10) 0.3 – 30  *y =* 0.0082 *x* + 0.0321 0.996 0.07

2H-O (1) 0.1 – 10  *y =* 0.0510 *x* + 0.1432 0.996 0.03

NO-O (1) 0.3 – 30  *y =* 0.0100 *x* +0.0097 0.994 0.15

**Stomach contents in case 1**

OLZ (1/10) 0.1 – 10  *y =* 0.1040 *x* + 0.1703 0.997 0.02

DM-O (1) 0.3 – 30  *y =* 0.0074 *x* + 0.0228 0.997 0.07

2H-O (1) 0.1 – 10  *y =* 0.0473 *x* + 0.0661 0.993 0.03

NO-O (1) 0.3 – 30 *y =* 0.0115 *x* + 0.0076 0.995 0.15

**Bile in case 1**

OLZ (1/100) 0.1 – 10 *y =* 0.1174 *x* + 0.2292 0.994 0.02

DM-O (1/100) 0.3 – 30  *y =* 0.0058 *x* + 0.0200 0.992 0.07

2H-O (1/10) 0.1 – 10  *y =* 0.0589 *x* + 0.1223 0.999 0.03

NO-O (1/10) 0.3 – 30 *y =* 0.0101 *x* + 0.0064 0.996 0.15

**Table S4** Intraday and interday accuracies/precisions, recoveries and matrix effects data for OLZ and its metabolites in spiked blood and urine samples using the matrix-matched calibration method

Intraday Interday Recovery Matrix effect

Analyte Conc.

Accuracy Precision Accuracy Precision

(ng/mL) (%) (% *RSD*) (%) (% *RSD*) (%) (% *RSD*) (%) (% *RSD*)

**Blood**

OLZ 0.05 96.8 10.2 108 8.7 　 81.5 11.0 81.6 6.7

1.0 93.3 3.6 95.1 3.0　　 95.5 　 8.0 87.3 　 5.0

10 89.7 1.7 93.3 2.5 89.6 13.2 91.3 2.0

DM-O 0.15 83.9 9.7 101 10.9 　 79.6 6.9 77.0 2.4

3.0 98.5 9.2 93.8 3.3　　 84.6 11.6 84.0 3.2

30 99.5 4.9 98.2 1.3 91.3 13.5 91.0 3.7

2H-O 0.05 92.4 12.9 88.0 6.8 　 82.8 10.7 81.0　　　8.8

1.0 111 1.5 111 9.0　　 87.9 9.3 98.0 　2.8

10 108 0.1 114 11.0 88.9 13.7 89.3 4.9

NO-O 0.15 106 10.1 111 11.2 　 75.9 6.9 83.0 4.1

3.0 99.4 4.3 93.4 5.2　　 84.9 6.1 90.6 5.0

30 102 9.7 98.7 5.0 93.1 0.2 95.0 1.6

**Urine**

OLZ 0.05 82.9 4.6 76.7 4.1 　 96.7　　　8.6　　　 79.7　　　2.9

1.0 114 2.2 118 7.1　　 83.3 5.2　　　 88.7　 3.5

10 99.9 1.4 99.8 3.4 86.0 8.8 93.3 7.6

DM-O 0.15 79.0 14.1 79.7 12.1 　 75.0 4.2 93.7 5.8

3.0 108 2.1 98.6 5.9　　 80.0 8.8 95.0 9.9

30 98.6 3.6 96.6 4.2 78.0 9.9 88.0 2.9

2H-O 0.05 75.6 5.5 84.9 4.1 　 91.0 2.8 98.7 10.5

1.0 105 0.55 108 5.4　　 107 10.6 104 8.2

10 103 1.3 101 12.0 98.3 　 5.6 78.7 9.8

NO-O 0.15 81.0 5.0 83.1 6.4 　 90.3 9.2 107 7.3

3.0 105 2.3 104 1.6　　 82.0 2.4 106 2.4

30 103 5.8 102 4.3 86.6 10.3 83.3 4.5

*RSD* relative standard deviation

**Table S5** Intraday (*n*=5) and interday (*n*=5) repeatability, recovery and matrix effect data for OLZ and its metabolites in pericardial fluid, stomach contents and bile in case 1 using the standard addition method

Analyte Intraday (*n*= 5) Interday (*n*= 5) Recovery Matrix effect

Concentration Repetability Concentration Repetability

(ng/mL) (% *RSD*) (ng/mL) (% *RSD*) (%±% *RSD*) (%±% *RSD*)

**Pericardial fluid**

OLZ 70.8 ±7.1 10.0 76.6 ±12.1 15.8 81.3 ±7.7 92.5±2.5

DM-O 39.6 ±3.4 8.6 35.2 ±7.2 20.4 83.7 ±12.7 90.1 ±9.1

2H-O 2.82±0.33 11.7 2.64±0.34 12.9 91.9±8.8 76.1±6.6

NO-O 0.97±0.08 8.2 1.10±0.26 23.6 96.7±13.2 85.3±10.0

**Stomach contents**

OLZ 16.9 ±0.7 4.1 16.4 ±2.5 15.2 89.6 ±3.7 83.9 ±5.1

DM-O 3.10 ±0.3 9.7 3.28 ±0.47 14.3 79.1±20.0 91.2 ±3.0

2H-O 1.40±0.22 15.7 1.44±0.23 16.0 83.4±3.2 79.6±2.98

NO-O 0.66±0.05 7.6 0.71±0.08 11.3 85.9±8.0 79.0±8.5

**Bile**

OLZ 200 ±18 9.0 182 ±24 13.2 84.3 ±11.9 84.2±9.5

DM-O 346 ±40 11.6 366 ±34 9.3 96.3 ±3.0 83.9 ±5.2

2H-O 20.8±2.3 11.1 22.0±2.3 10.5 94.8±2.8 86.0±14.4

NO-O 6.30±1.07 17.0 6.12±1.02 16.7 78.0±8.6 81.6±6.5
